# Supplementary material for: MCSPACE: inferring microbiome spatiotemporal dynamics from high-throughput co-localization data
Source: Microbiome. 2025 Dec 12;14:28. doi: 10.1186/s40168-025-02279-4 (PMC12817806; doi:10.1186/s40168-025-02279-4)
Supplement: Supplementary file 2 — Additional file 1: Table S1. Composition of diets used in mouse study, in g/Kg. Fig. S1: SAMPL-seq data pre-processing filtering curves. Thresholds for minimum reads per particle and OTU abundance across particles were varied, with the number of retained particles and OTUs plotted against each threshold. (A-C) Number of OTUs remaining versus minimum relative abundance thresholds for human (A), human with inulin perturbation (B), and mouse (C) datasets. (D-F) Number of particles remaining versus threshold for minimum number of reads per particle for human (D), human with inulin perturbation (E), and mouse (F) datasets. Red circles and corresponding red value on x-axis correspond to estimates of “elbows” (0.005 minimum abundance and 250 minimum reads per particle), which were then used for final data filtering. Fig. S2. Summary of longitudinal human SAMPL-seq spatial co-localization dataset. Visualization of filtered data from a longitudinal study of gut microbiome spatial co-localization in a healthy human participant (collected daily for five days, n = 5 fecal samples total). (A) Phylogenetic tree of Operational Taxonomic Units (OTUs) present in particles. (B) Clustered heatmap of particles, showing the relative abundance of taxa in particles over each of the five consecutive days. (C) Density plots of OTUs per particle. Fig. S3. Summary of longitudinal human SAMPL-seq spatial co-localization dataset with inulin supplementation. Visualization of filtered data from a longitudinal study of gut microbiome spatial co-localization in a healthy human participant with inulin supplementation perturbation (collected over 12 days, n = 7 fecal samples total). (A) Phylogenetic tree of Operational Taxonomic Units (OTUs) present in particles. (B) Clustered heatmap of particles, showing the relative abundance of taxa in particles in each sample. (C) Density plots of OTUs per particle. Fig. S4. Summary of new murine longitudinal SAMPL-seq spatial co-localization dataset with multip [file 40168_2025_2279_MOESM1_ESM.docx]

**Supplementary Tables:**

**Supplementary Table 1: Composition of diets used in mouse study, in g/Kg.**

|  | Standard | High fat | High fat, high fiber | Low protein |
| --- | --- | --- | --- | --- |
| Casein | 210 | 265 | 265 | 69 |
| L-cystine | 3 | 4 | 4 | 0 |
| DL-Methionine | 0 | 0 | 0 | 0.9 |
| Lard | 20 | 310 | 310 | 0 |
| Soybean oil | 20 | 30 | 30 | 0 |
| Corn oil | 0 | 0 | 0 | 53.9 |
| Corn starch | 465 | 0 | 0 | 200 |
| Maltodextrin | 100 | 160 | 160 | 0 |
| Sucrose | 90 | 90 | 55.5 | 571.7 |
| Cellulose | 37.15 | 65.5 | 0 | 57.82 |
| Inulin | 0 | 0 | 50 | 0 |
| Pectin | 0 | 0 | 50 | 0 |

**Supplementary Figures:**


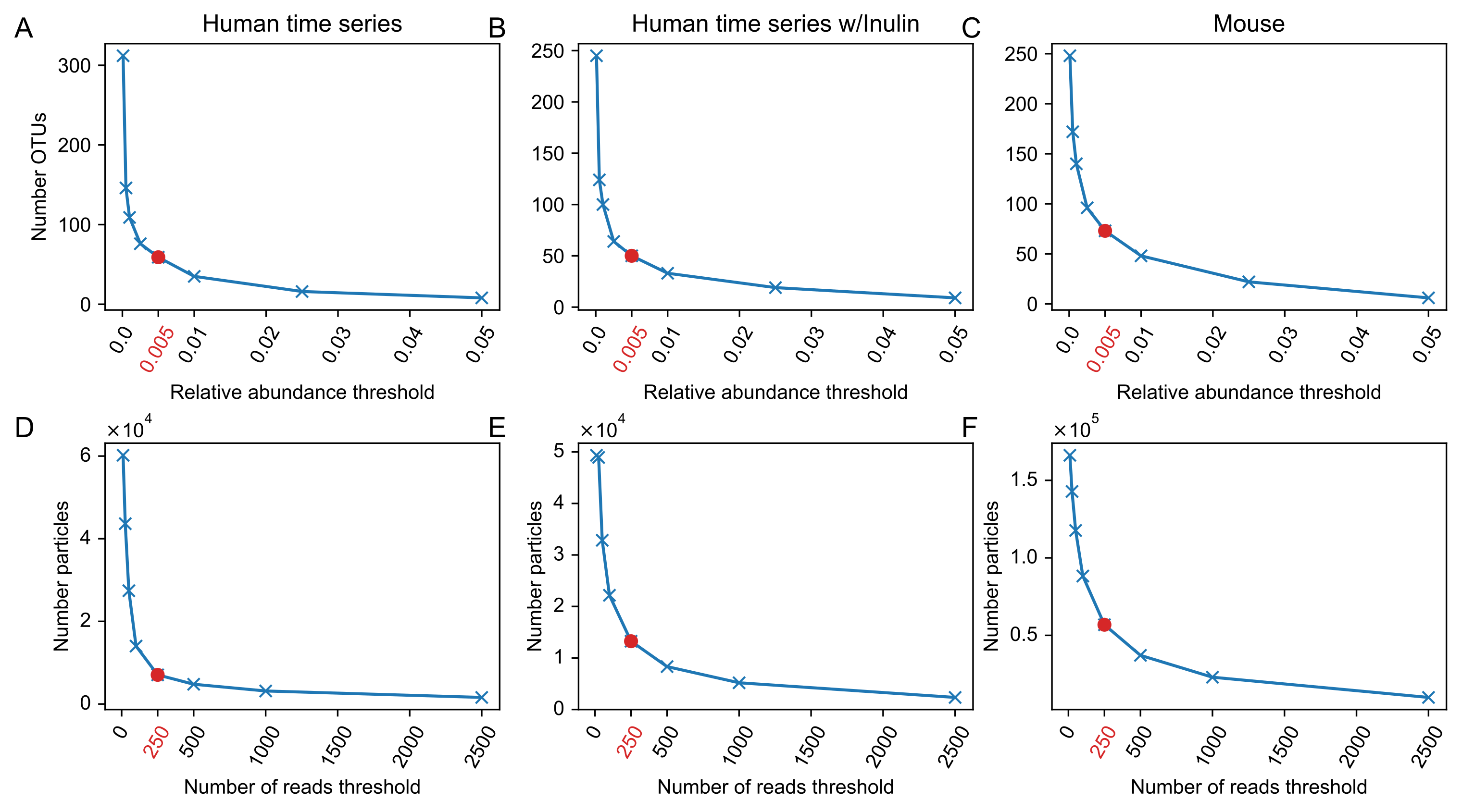


**Supplementary Figure 1: SAMPL-seq data pre-processing filtering curves.** Thresholds for minimum reads per particle and OTU abundance across particles were varied, with the number of retained particles and OTUs plotted against each threshold. **(A-C)** Number of OTUs remaining versus minimum relative abundance thresholds for human **(A)**, human with inulin perturbation **(B)**, and mouse **(C)** datasets. **(D-F)** Number of particles remaining versus threshold for minimum number of reads per particle for human **(D)**, human with inulin perturbation **(E)**, and mouse **(F)** datasets. Red circles and corresponding red value on x-axis correspond to estimates of “elbows” (0.005 minimum abundance and 250 minimum reads per particle), which were then used for final data filtering.


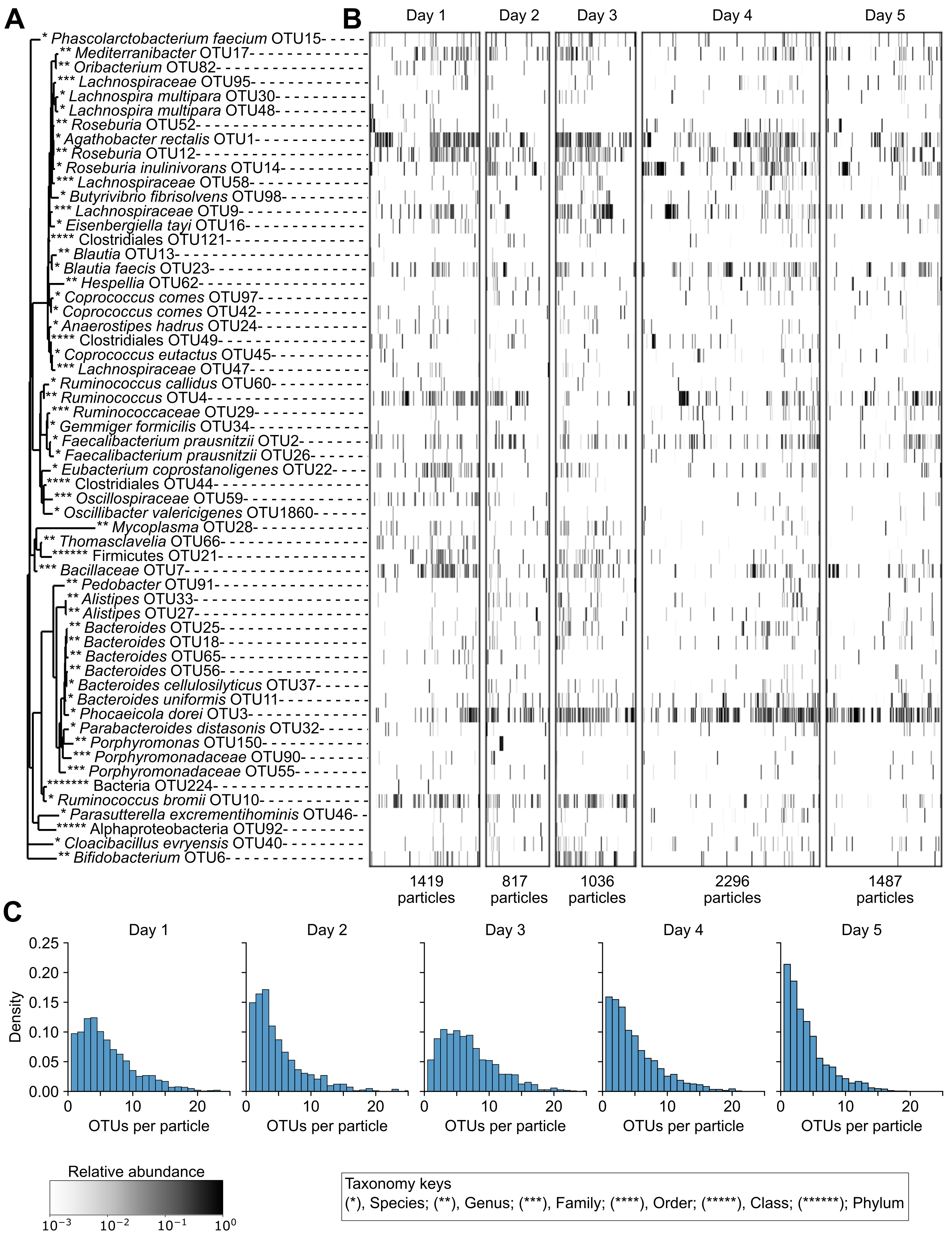


**Supplementary Figure 2: Summary of longitudinal human SAMPL-seq spatial co-localization dataset.** Visualization of filtered data from a longitudinal study of gut microbiome spatial co-localization in a healthy human participant (collected daily for five days, n=5 fecal samples total). **(A)** Phylogenetic tree of Operational Taxonomic Units (OTUs) present in particles. **(B)** Clustered heatmap of particles, showing the relative abundance of taxa in particles over each of the five consecutive days. **(C)** Density plots of OTUs per particle.


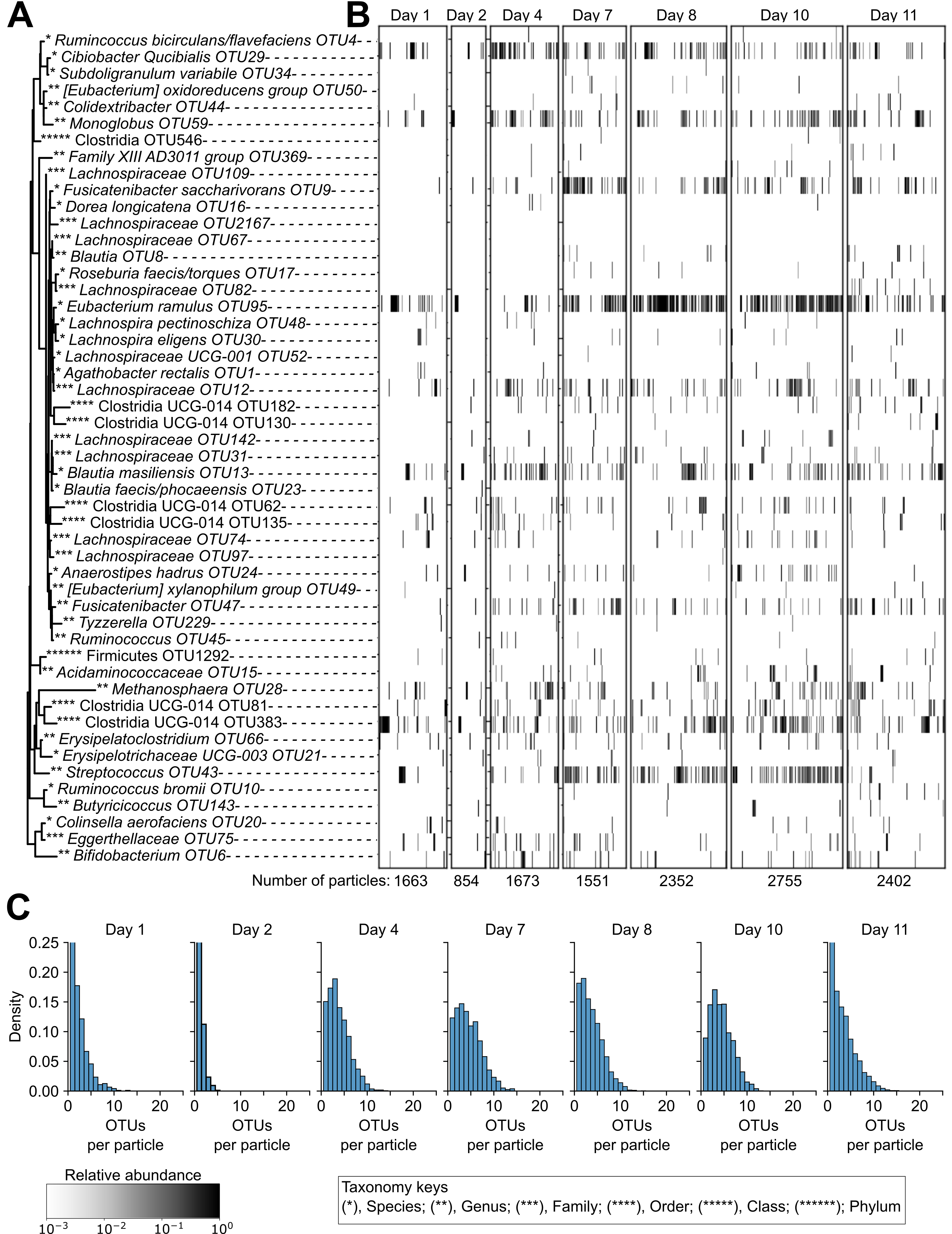


**Supplemental figure 3: Summary of longitudinal human SAMPL-seq spatial co-localization dataset with inulin supplementation.** Visualization of filtered data from a longitudinal study of gut microbiome spatial co-localization in a healthy human participant with inulin supplementation perturbation (collected over 12 days, n=7 fecal samples total). **(A)** Phylogenetic tree of Operational Taxonomic Units (OTUs) present in particles. **(B)** Clustered heatmap of particles, showing the relative abundance of taxa in particles in each sample. **(C)** Density plots of OTUs per particle.


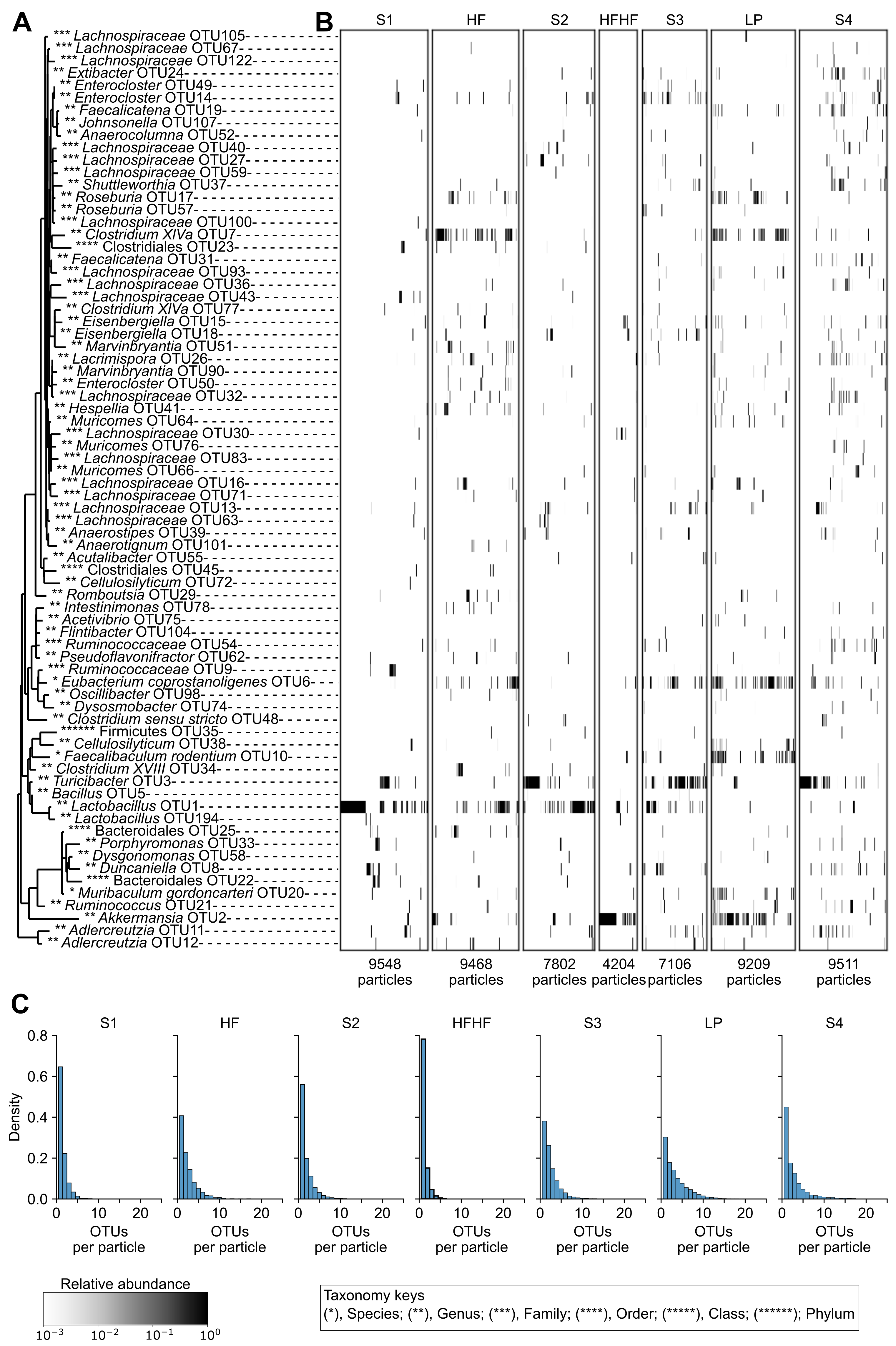


**Supplementary Figure 4:** **Summary of new murine longitudinal SAMPL-seq spatial co-localization dataset with multiple dietary perturbations.** Visualization of filtered data from longitudinal study of gut microbiome spatial co-localization in 3 mice subjected to defined dietary perturbations (n=21 total fecal samples). HF = high fat; HFHF = high fat, high fiber; LP = low protein; S1-4 = standard diet 1-4. **(A)** Phylogenetic tree of OTUs present in particles. **(B)** Hierarchically clustered heatmap of particles, pooled over the three mice, showing the relative abundance of taxa in particles in each of the diets. **(C)** Density plots of OTUs per particle distributions, pooled over the three mice, for each diet.


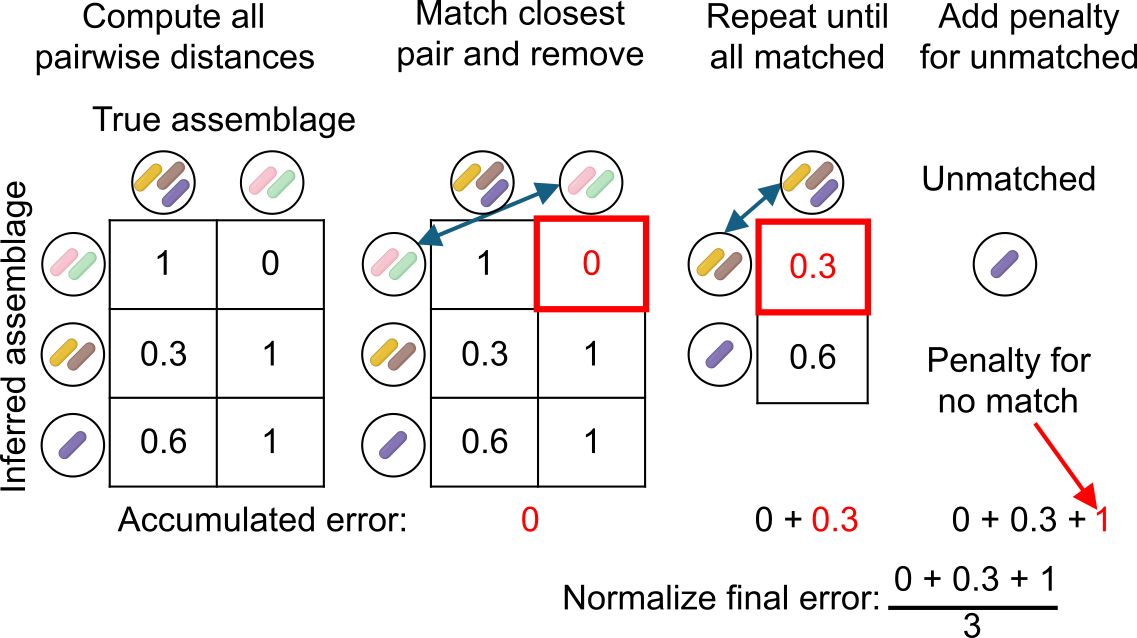


**Supplementary Figure 5: Schematic of assemblage recovery error metric calculation**. Our metric accounts for differences in both the number and order of recovered assemblages, which may differ from that of ground truth. To compute the metric, a greedy algorithm matches inferred and true assemblages by iteratively pairing the closest matches based on pairwise distances, then accumulating errors for each pair. Unmatched assemblages incur a penalty, and the total error is normalized by the larger of the inferred or true assemblage count. Full algorithmic details are provided in the **Methods** section.


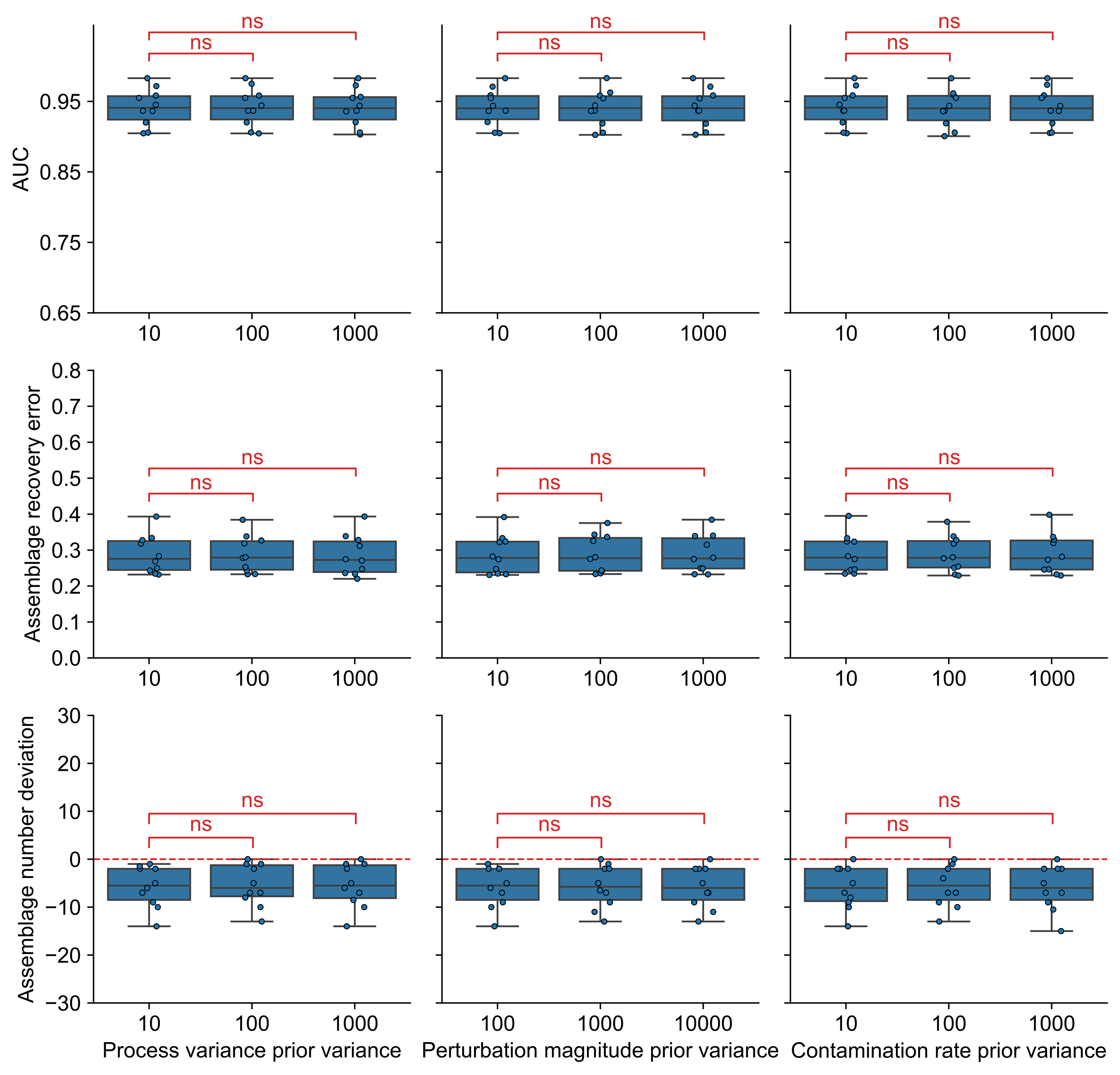


**Supplemental Figure 6: MCSPACE performance is robust to hyperparameter settings.** MCSPACE was evaluated using default settings and with hyperparameters for the variance on the prior for process variance, perturbation magnitude, and contamination weight increased by 10X and 100X. Benchmarking metrics were consistent across all settings, indicating that model performance is robust to the chosen hyperparameters. Semi-synthetic data was simulated from the MCSPACE model inferred on real data. Methods were assessed for their ability to recover ground-truth information in three tasks: **(Top row)** Detecting co-associated pairs of microbes, assessed with area under the receiver operator curve (AUC) (higher values indicate superior performance), **(Middle row)** Recovering the correct frequencies of OTUs in assemblages, assessed using assemblage recovery error (lower values indicate superior performance), and **(Bottom row)** Inferring the correct number of assemblages, assessed by subtracting the inferred value from the correct value (values nearer to zero indicate superior performance). Boxplots show results from 10 simulated replicates. Central lines indicate medians with boxes representing interquartile ranges (IQRs). Whiskers extend to data points within 1.5 × IQR. Statistical significance was assessed with a Wilcoxon rank sum test followed by Benjamini-Hochberg correction for multiple hypothesis testing*.*


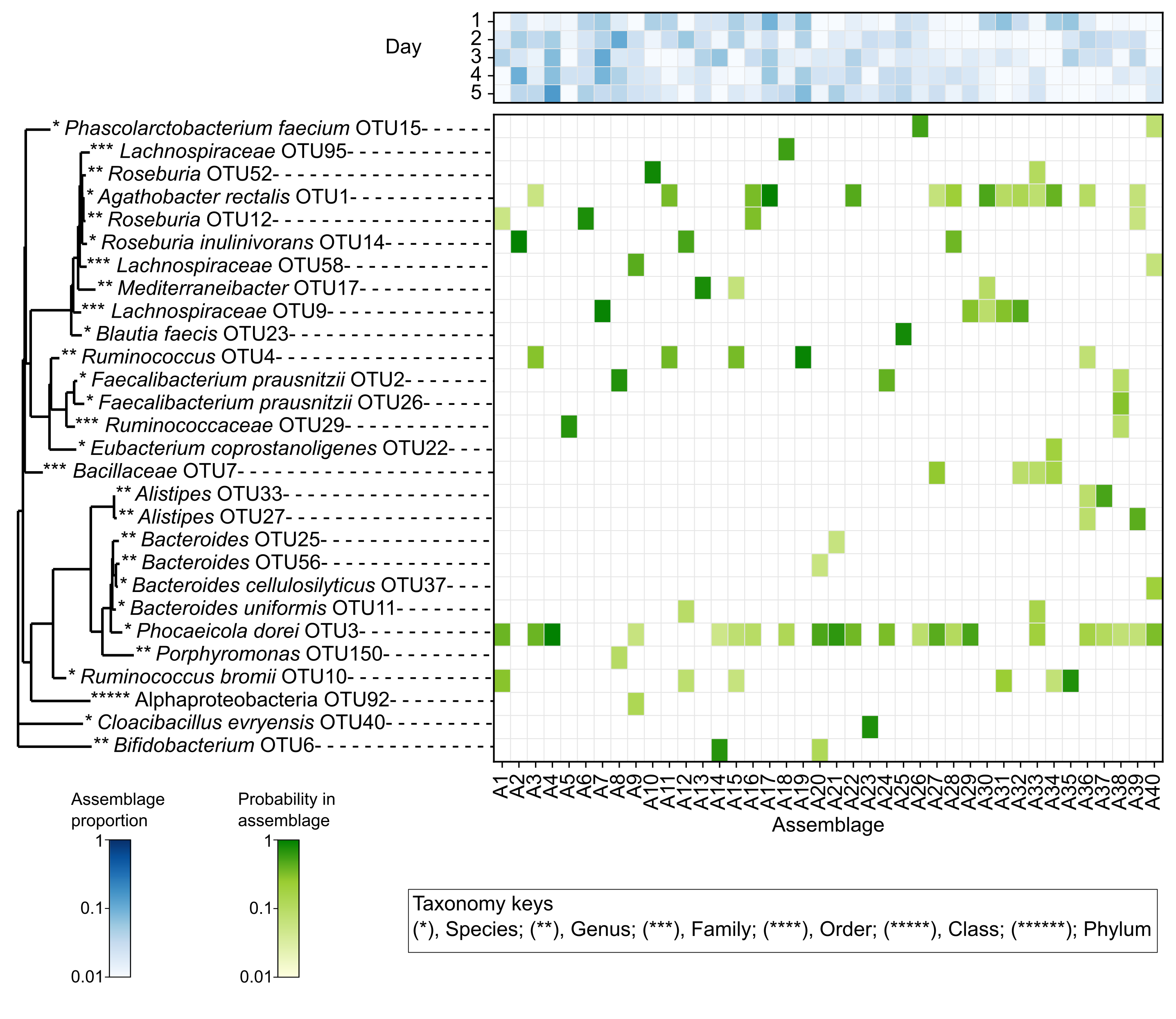


**Supplementary Figure 7: MCSPACE identified spatial assemblages among taxa and temporal changes in assemblage proportions in the human gut microbiome from a longitudinal SAMPL-seq dataset.** MCSPACE identified 58 OTUs assorting into 40 spatial assemblages, with assemblage abundances tracked over time. A phylogenetic tree of OTUs present in the dataset is displayed on the left. Heatmaps show assemblage proportions over the five consecutive days in the study (above), and OTU frequencies in inferred spatial assemblages (below) with OTU assemblage frequencies ≥0.05 shown.


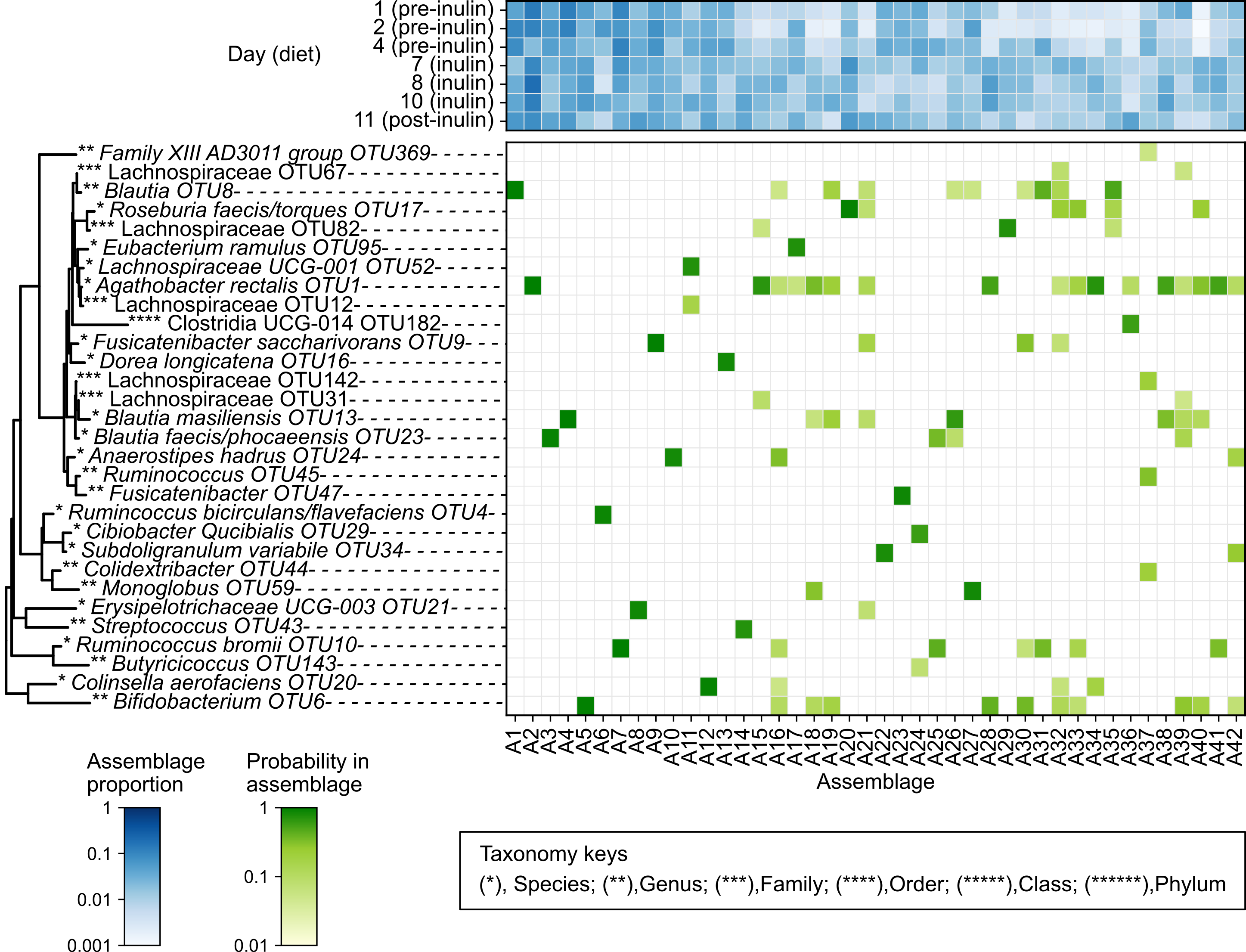


**Supplemental figure 8: MCSPACE identified spatial assemblages and their temporal changes in SAMPL-seq human gut microbiome dataset with inulin perturbation.** MCSPACE identified 50 OTUs assorting into 42 spatial assemblages, with assemblage abundances tracked over time. A phylogenetic tree of OTUs present in the dataset is displayed on the left. Heatmaps show assemblage proportions over each day in the study (above), and OTU frequencies in inferred spatial assemblages (below) with OTU assemblage frequencies $\geq$0.05 shown.


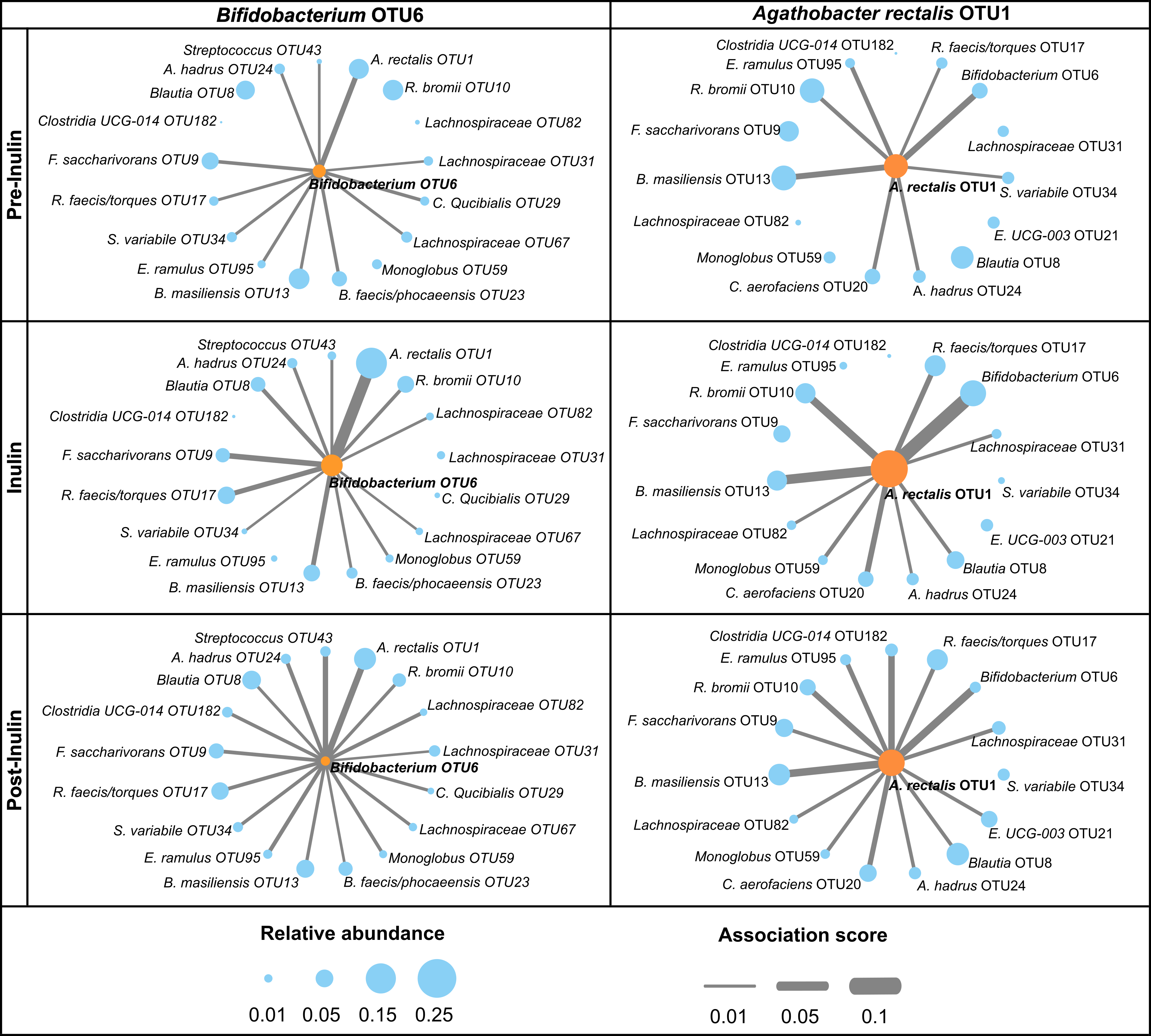


**Supplemental figure 9: MCSPACE analysis reveals inulin supplement-induced changes in spatial associations of key taxa in human study.** Spatial association mapping of Bifidobacterium OTU6 and Agathobacter rectalis OTU1. Maps depict associations on pre-inulin, inulin, and post-inulin supplementation dietary intervals. Node sizes indicate relative abundances of taxa and edge widths indicate the strengths of spatial associations (<0.01 not shown). Relative abundances and spatial association scores were averaged over days in each dietary period (days 1,2,4 for pre-inulin; 7,8,10 for inulin; 11 for post-inulin). During inulin supplementation, Bifidobacterium OTU6 gained or strengthened associations with many taxa (e.g. A. rectalis OTU1, R. faecis/torques OTU17, Blautia OTU8, Lachnospiraceae OTU82, and F. saccharivorans OTU9), many of which also increased in abundance during inulin. Several associations persisted post-inulin. A. rectalis OTU1 also gained associations during and after inulin supplementation, including a transient increase in its association with Bifidobacterium OTU6.


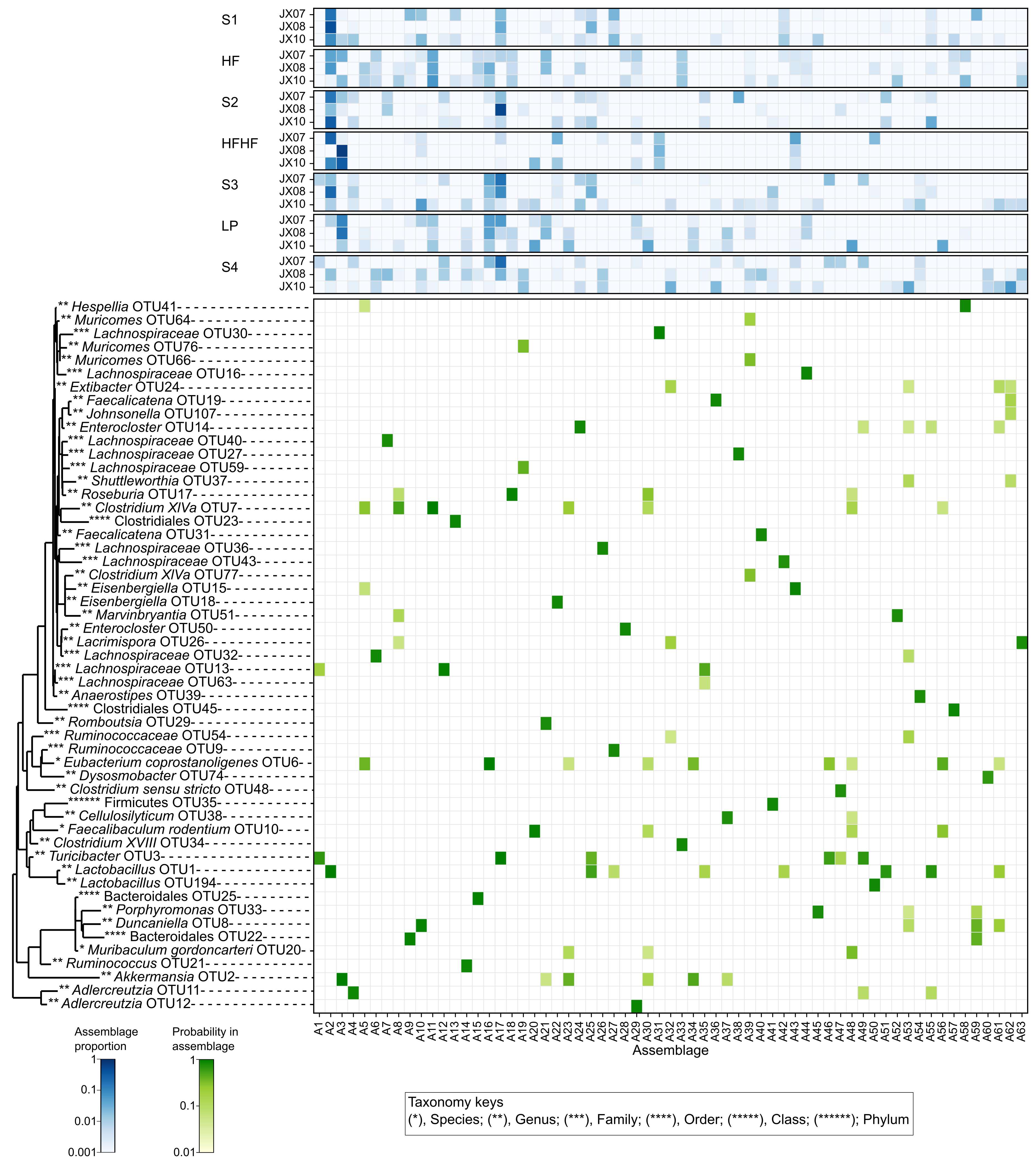


**Supplementary Figure 10: MCSPACE identified spatial assemblages among taxa and changes in assemblage proportions in the murine gut microbiome from a SAMPL-seq dataset investigating multiple dietary perturbations.** MCSPACE identified 74 OTUs assorting into 63 spatial assemblages, with assemblage abundances tracked over time for three biological replicates. A phylogenetic tree of OTUs present in the dataset is shown on the left. Heatmaps show assemblage proportions (above) inferred for each biological replicate over the seven dietary intervals (HF = high fat; HFHF = high fat, high fiber; LP = low protein; S1-4 = standard diet 1-4), and OTU frequencies in inferred spatial assemblages (below). OTUs with assemblage frequencies *≥*0.05 are shown.


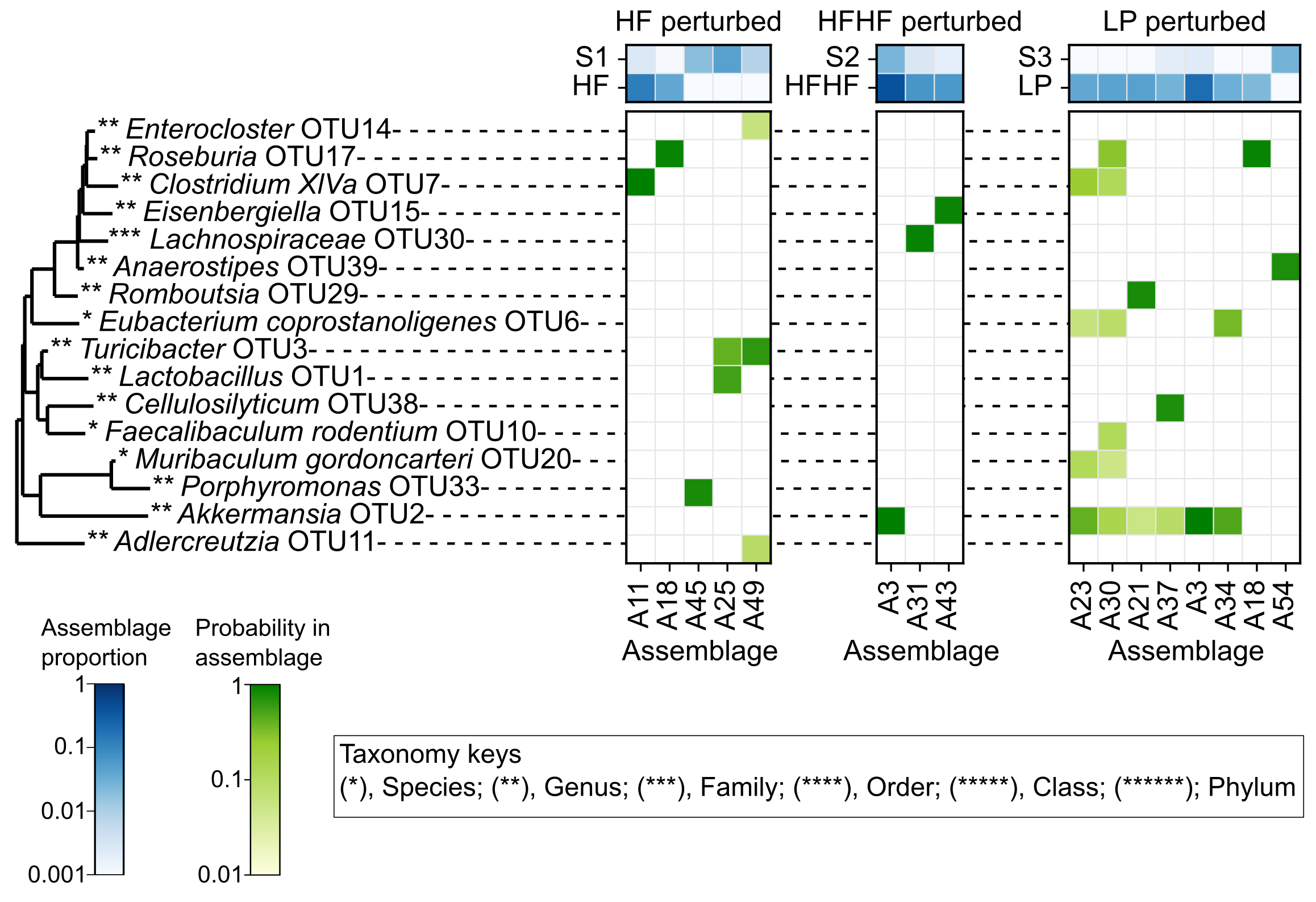


**Supplementary Figure 11: MCSPACE analysis of the murine data revealed diet-induced spatial shifts in the gut microbiome.** Assemblages with strong evidence of an effect from one or more perturbations (Bayes Factors > 10) are shown for each dietary perturbation. HF = high fat; HFHF = high fat, high fiber; LP = low protein; S1-3 = standard diet 1-3. Phylogenetic tree of OTUs present with frequency >5% in any of the perturbed assemblages is shown on the left. Heatmaps show assemblage proportions before and after perturbation for each dietary perturbation (above) and OTU frequencies in perturbed spatial assemblages (below).
